# Empowered citizen ‘health hackers’ who are not waiting

**DOI:** 10.1186/s12916-016-0670-y

**Published:** 2016-08-17

**Authors:** Timothy Omer

**Affiliations:** London, UK

**Keywords:** Type 1 diabetes, Artificial pancreas, Citizen Hackers, #WeAreNotWaiting

## Abstract

Due to the easier access to information, the availability of low cost technologies and the involvement of well educated, passionate patients, a group of citizen ‘Health Hackers’, who are building their own medical systems to help them overcome the unmet needs of their conditions, is emerging. This has recently been the case in the type 1 diabetes community, under the movement #WeAreNotWaiting, with innovative use of current medical devices hacked to access data and Open-Source code producing solutions ranging from remote monitoring of diabetic children to producing an Artificial Pancreas System to automate the management and monitoring of a patient’s condition. Timothy Omer is working with the community to utilise the technology already in his pocket to build a mobile- and smartwatch-based Artificial Pancreas System.

## Background

Type 1 diabetes is a condition requiring around the clock management. Despite the multiple daily injections and invasive blood testing, type 1 diabetes patients find that the main challenge to managing their condition is the mental pressure to keep track of their blood sugar levels, treatments and medication calculations, leading them to exhaustion. After more than 22 years as a type 1 diabetic, I agree with them.

### Existing care is unrealistic

The NHS Type 1 Diabetes Care Pathway [1] is focused on a yearly follow-up between patients and a diabetes clinic of trained experts. Patient performance over the previous year is reviewed and treatment adjustments suggested, but this is often of limited value for the patient, whose state on a given day may often not match that on the next, and a yearly discussion cannot summarise the patient’s personal challenges. Therefore, the outcome is often a ‘bending of the truth’ to meet the clinician’s expectations.

The most modern accessible technology for type 1 diabetes management is an insulin pump, which provides a constant supply of insulin, as well as a self-funded [2] continuous glucose monitor, which provides real-time feedback of the patient’s blood sugar levels. These devices provide many functions and high volumes of data, all of which are very welcome and useful, but such systems always fail with regards to patient expectations to understand and process all of this information. As a result, patients become overwhelmed by a feeling of judgement by healthcare professionals, the vast amounts of ensuing information, and alert and alarm ‘shouts’ from their devices when they have failed at being a ‘good diabetic’, as well as with their own disappointment of their body letting them down.

### Are things going to get better?

Diabetics are getting better at understanding their condition and its cause and reactions, as well as more technically able to finely tune treatments and understand real-time information, yet patients are still not able to processes and act upon such data; this is due to incorrect use of the data. Data is commonly locked into the devices, with no or limited ability to share across devices, and the analytics required to process such data are extremely complicated. Nevertheless, patients should not be made to feel that managing their diabetes is their full-time job!

### When enough is enough

The lack of accessible and actionable data is a common frustration in the type 1 diabetes community. Given the easier communication between diabetics with online communities in recent times, the community have been engaging in conversation, with a key event in the community being the first DiabetesMine D-Data ExChange event [3], which highlighted the frustration of patients waiting for their needs to be addressed and where the community declared “We Are Not Waiting”. And rightly so, why should patients wait to address their current needs?

Patients currently have access to global online communities that allow unrestricted sharing of ideas, projects and collaboration efforts to overcome challenges [4], as well as access to cheap technology from the Hobby Electronics movement [5] and a passionate well-educated pool of individuals with a common goal. This critical mass, which rallied under the hashtag #WeAreNotWaiting, saw rapid progress in DIY medical devices in the type 1 diabetes community [6].

### Patient-driven projects

Through community-driven innovation there has been a surge in edge cases being developed, which may be of little interest to the medical industry either due to costs or risks. By gaining access to their own data under their terms, several patient-driven creative solutions have been developed:

- To monitor continuous glucose monitor data from a smartwatch [7]
- To remotely monitor a child’s blood sugar levels [8]
- To provide louder alarms and treatment suggestions according to the data [9]
- To analyse patient data and automate medication delivery [10]

These projects are empowering the patient through the better use of real-time data to help manage a real-time condition. Watching one’s blood sugars rise on a smartwatch after eating a pizza is a lot more powerful than the screen on the manufactures device showing the same data in one’s bag.

### The path to a DIY artificial pancreas

Only a year ago, the thought of an artificial pancreas system (APS) was overwhelming. The multiple components required and skill needed to provide an efficient and safe system is beyond the majority of citizens (with some exceptions [11]), yet the individual challenges are more easily tackled. Through a step-by-step method of collecting, merging and processing community accessed data from patients’ devices to provide meaningful actionable information, there has been a surge in the sharing and convergence of community DIY projects. One of the outcomes is the OpenAPS project [8], which provides the instructions and blueprint of a DIY patient-built APS. Yet, how effective can a patient-built ‘amateur’ system be? This is best summed up by a quote from a recent OpenAPS user:

“*Dear Machines: You Can Take This Job*” [12]

### Rise of the machines

In 2015, I decided to fork (make a copy of) the OpenAPS project and make use of the supercomputer in my pocket to assist with management of my diabetes. With the communities Open Source projects, namely xDrip and OpenAPS, I self-taught myself mobile development and built an APS app [13]. I had no need to start from scratch or learn the best algorithm to crunch my data, I took what the community had already developed to kick start my own project, which allowed me to focus on building a system unique to my needs and on the additional functionality that I required.

While the system I built was an Open Loop system lacking communication with my insulin pump, this was enough to significantly help me with the management and treatment of my condition. The system assisted with one of the most problematic areas of my care – me. The app is free from the frustration, impatience and, at times, simple human ignorance, it does what I cannot – analyse my data every 5 minutes and make an unemotional logical decision. I moved from reactive to proactive management of my diabetes, where the APS system would provide treatment alterations to manage the highs and lows of my blood sugar levels and often stop such events from occurring. It is a liberating feeling, for the first time in 22 years I can let my diabetes take a back seat without damaging my health.

### The data-rich patient

Where is this heading? Access to information, collaboration and low technology costs are only going to improve over time. As the communities’ expertise improves, there will be an increase in medical management challenges tackled by the community. Patients will be able to understand their conditions by analysing the wealth of information captured, access rapid production technologies leading to easily available high quality tools [14], and be on a par with the technically capable 1 % due to more accessible tools and sharing of knowledge through community-led workshops [15]. This will spread beyond diabetes care to other conditions ripe for such disruption [16].

### Regulate the unregulatable?

Such rapid innovation and progress in the community is partly the result of a lack of regulation. As with all medical devices, there is a risk of their inappropriate use or misuse. DIY medical devices are not for all, but the work of the community should not be categorised by just the more ambitious projects out there. Community projects could be simply categorised as (1) low risk (e.g. gatherings and workshops discussing diabetes management, DIY projects that visualise and access patient data in more meaningful ways, and 3D printed components to help organise and arrange equipment); (2) medium risk (e.g. DIY projects providing automated suggestions on treatment adjustments); and (3) high risk (DIY projects providing automated monitoring and delivery of medication).

The progress of patient-led innovation shows no sign of slowing and ignoring such a movement will not only be futile but also a lost opportunity in the way care is provided for such long-term conditions. Community work should not be seen as competition to the medical industry or healthcare providers, but rather as a treatment path challenging and pushing the boundaries of what is currently possible, as well as being a wealth of free data [17]. Categorising the type of community care that can be provided will allow assessment by the medical industry and healthcare providers for them to decide on what they are willing to support and what they would prefer to be less involved in. With the United Kingdom’s National Health Service in turmoil due to spiralling costs, here lies an opportunity to utilise a pool of human resources with a proactive interest in their condition and a willingness to share their knowledge with others. These are the empowered patients who own their condition – support and encouragement of such communities can be provided at almost zero cost to the National Health Service. It is time to start embracing this community and acknowledging their potential as well as the possible risks that some projects may introduce.

## Conclusions

The community is a large, experienced and skilled resource that is currently underused. Their access to information, sharing of ideas and drive to improve their quality of life will continue to flourish [18]. Do these DIY projects really improve the management of chronic conditions? Or is this just an effect of the patient being more active in their management? For me, the outcome is the same, I am empowered by the community and the tools they have developed. We are now managing our condition and, whether you agree or not, progress is not slowing, as ‘We Are Not Waiting’.
